# Immune-mediated lung diseases: A narrative review

**DOI:** 10.3389/fmed.2023.1160755

**Published:** 2023-04-06

**Authors:** Jaleel Jerry G. Sweis, Nabil W. G. Sweis, Fatima Alnaimat, Jacqueline Jansz, Ting-Wei Ernie Liao, Alaa Alsakaty, Abeera Azam, Hesham Elmergawy, Hali A. Hanson, Christian Ascoli, Israel Rubinstein, Nadera Sweiss

**Affiliations:** ^1^School of Medicine, The University of Jordan, Amman, Jordan; ^2^Division of Rheumatology, Department of Internal Medicine, The University of Jordan, Amman, Jordan; ^3^Department of Medicine, University of Illinois Chicago, Chicago, IL, United States; ^4^School of Medicine, Faculty of Medicine, National Yang Ming Chiao Tung University, Taipei City, Taiwan; ^5^Division of Cardiology, Department of Medicine, Taipei Veterans General Hospital, Taipei City, Taiwan; ^6^Division of Rheumatology, Department of Medicine, University of Illinois Chicago, Chicago, IL, United States; ^7^Department of Internal Medicine, The University of Texas Health Science Center at Tyler, Tyler, TX, United States; ^8^UIC College of Pharmacy, University of Illinois Chicago, Chicago, IL, United States; ^9^Division of Pulmonary, Critical Care, Sleep and Allergy, Department of Medicine, University of Illinois Chicago, Chicago, IL, United States; ^10^Research Service, Jesse Brown VA Medical Center, Chicago, IL, United States

**Keywords:** immune-mediated lung diseases, interstitial lung disease, connective tissue diseases, post-COVID-19, idiopathic pulmonary fibrosis, sarcoidosis, drug-induced lung injury, post-lung transplant

## Abstract

The role of immunity in the pathogenesis of various pulmonary diseases, particularly interstitial lung diseases (ILDs), is being increasingly appreciated as mechanistic discoveries advance our knowledge in the field. Immune-mediated lung diseases demonstrate clinical and immunological heterogeneity and can be etiologically categorized into connective tissue disease (CTD)-associated, exposure-related, idiopathic, and other miscellaneous lung diseases including sarcoidosis, and post-lung transplant ILD. The immunopathogenesis of many of these diseases remains poorly defined and possibly involves either immune dysregulation, abnormal healing, chronic inflammation, or a combination of these, often in a background of genetic susceptibility. The heterogeneity and complex immunopathogenesis of ILDs complicate management, and thus a collaborative treatment team should work toward an individualized approach to address the unique needs of each patient. Current management of immune-mediated lung diseases is challenging; the choice of therapy is etiology-driven and includes corticosteroids, immunomodulatory drugs such as methotrexate, cyclophosphamide and mycophenolate mofetil, rituximab, or other measures such as discontinuation or avoidance of the inciting agent in exposure-related ILDs. Antifibrotic therapy is approved for some of the ILDs (e.g., idiopathic pulmonary fibrosis) and is being investigated for many others and has shown promising preliminary results. A dire need for advances in the management of immune-mediated lung disease persists in the absence of standardized management guidelines.

## 1. Introduction

The immune system is a heterogeneous collection of physical, cellular, and biochemical components that defend against microbial organisms and non-microbial foreign antigens. Based on their antigen specificity and promptness of response, the immune system’s components can be divided into innate and adaptive immune arms (1). Innate immunity represents the early response system to foreign body entry. It comprises cutaneous and mucosal barriers, phagocytic cells (e.g., macrophages and neutrophils), soluble factors (e.g., complement system), and receptors (e.g., pattern recognition receptors). The rapidity of the response of the innate arm is a crucial feature conferred by the capacity of its receptors to recognize pre-determined structural patterns common to a vast array of foreign pathogens, which accounts for its lack of specificity (2). The delayed, more antigen-specific adaptive immune response ensues, generating cells and/or mediators specifically tailored to the invading pathogen or substance, producing a more efficient response. The adaptive immune system can be further divided into humoral immunity, effectuated by B lymphocytes and their secreted antibodies, and cellular immunity, effectuated by various subsets of T lymphocytes. The adaptive response also generates memory cells for a more rapid anamnestic response against the same foreign antigen should re-exposure occur (3). As convenient as the classification may seem, the innate and adaptive arms are not mutually exclusive; a substantial degree of crosstalk occurs between both systems *via* cells (e.g., dendritic cells) and an intricate collection of mediators orchestrates and fine-tunes the immune response (4).

The respiratory tract merits special consideration in the discussion of immunity. By virtue of its gas exchange function, the respiratory tract is a stage for constant clashes between the immune system and foreign exposures that manage to trickle into the airways with each breath (4). As such, the respiratory system is equipped with a remarkable set of defense lines, from the mucociliary apparatus and respiratory epithelium to the effector immune cells lingering in pulmonary tissue. Small particles that overcome the mechanical barrier are initially faced with resident innate immune cells, namely, the alveolar macrophages, alongside a group of secreted enzymes, immunoglobulins, and antimicrobial factors (5). Recruitment of other cells from the innate and adaptive systems occurs for more effective clearance of the foreign substance. Despite affording great protection to the lungs in particular and the body in general, the respiratory immune system may prove detrimental in conditions where it is dysregulated or generates an exaggerated response. In such settings, a wide range of pulmonary pathologies can emerge and have been described quite extensively (6). Nevertheless, the specific mechanisms that link immunity to lung injury in many of these disease entities remain poorly defined and are integral to devising targeted therapeutic interventions, especially for the diffuse parenchymal lung diseases.

Diffuse parenchymal lung diseases, also referred to as interstitial lung diseases (ILDs), are a diverse set of illnesses grouped according to comparable clinical, radiological, physiological, or pathologic characteristics (7–9). The word “interstitial” is used to describe the pathologic appearance that the aberration starts in the interstitium. Still, it is slightly misleading because most of these disorders are also linked to significant changes in the alveolar and pulmonary architecture. Among the various ILDs, sarcoidosis, connective tissue disease (CTD)–associated ILDs, and idiopathic pulmonary fibrosis (IPF) are the most common fibrotic ILDs, with an estimated prevalence of 30.2, 12.1, and 8.2 cases per 100,000, respectively (10). A multidisciplinary approach, typically involving pulmonologists, radiologists, and pathologists, is necessary for the differential diagnosis of ILDs (11). Clinical presentation, a thorough history review (medications, infectious and occupational exposures), smoking status, changes in lung function, the findings of serological tests, imaging, and, if necessary, a lung biopsy are all part of an evaluation (9, 12, 13). ILD is first suspected when a patient experiences progressive shortness with exertion (dyspnea), a prolonged non-productive cough, and/or pulmonary symptoms linked to another illness, including rheumatic disease. Regular laboratory testing is frequently non-specific. High-resolution computed tomography (HRCT) of the chest is the primary diagnostic tool for ILD (13). The underlying diagnosis and the progression of the disease serve as the basis for decisions regarding pharmacologic treatment. Antifibrotic medications, such as pirfenidone or nintedanib, are indicated for IPF patients (14). In most cases of fibrosing ILD other than IPF, immunomodulation with glucocorticoids, immunosuppressive therapy, or both are appropriate and are often utilized as first-line therapy if there is a suspicion of inflammation-driven disease (15–17).

**FIGURE 1:**
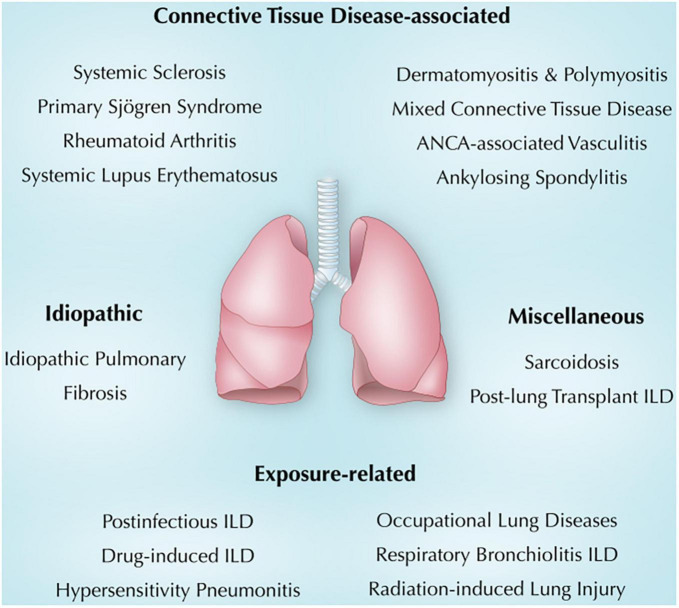
Overview of immune-mediated lung diseases discussed in this review. ANCA, anti-neutrophil cytoplasmic antibodies; ILD, interstitial lung disease.

The association between ILD and immunity in general and autoimmunity in particular is evolving. This review summarizes the current understanding of immunity and lung injury observed in CTD-associated, exposure-related, idiopathic, and other miscellaneous lung diseases (Figure 1). Also, we will shed light on common clinical, radiologic, and pathologic findings in each section. It is crucial to make an early diagnosis, identify triggers and consider immunosuppressive therapy when indicated (6). Other ILDs, including idiopathic interstitial pneumonia and ILDs with cysts and/or airspace filling (18), were not discussed in this review.

## 2. Connective tissue disease-associated lung diseases

Connective tissue disease -associated lung diseases are summarized in Table 1.

**TABLE 1:** Summary of CTD-associated lung diseases.

	Systemic sclerosis	Primary Sjögren syndrome	Rheumatoid arthritis	Systemic lupus erythematosus (SLE)	Dermatomyositis (DM) and polymyositis (PM)	MCTD	Systemic (ANCA-associated) vasculitis	Ankylosing pondylitis
Clinical manifestations	Raynaud’s phenomenon, MSS (arthralgia or arthritis), skin (tightening, thickening), GIS, RS (ILD, PAH), renal system (renal crisis), CVS (arrhythmia, pericardial disease, HF).	Dry eyes, mouth, and mucous membrane, parotitis, MSS (arthritis), parotitis; RS (ILD), skin, renal system, CNS, lymph nodes may rarely be affected	MSS (most common), rarely RS (ILD) and vascular system (vasculitis), skin (pyoderma, rheumatoid nodules), eyes (scleritis, keratitis)	MSS, renal system (lupus nephritis), skin, mucous membranes, RS (ILD), CVS (pericardial disease, MI), CNS (seizures, psychosis), hematological abnormalities (cytopenia)	MSS (proximal muscle weakness), skin in DM (Symmetric erythematous rash), RS (ILD), CVS (myocarditis, HF). Increased risk of malignancy	Raynaud’s phenoumenon; MSS (arthralgia, myalgia), RS (ILD, PAH), CVS (pericarditis), Renal system, and CNS may be affected	Vascular system (wall thickening), skin (purpura), RS (ILD), CNS (headache, seizures, ischemia), MSS (arthralgia), renal system (glomerulonephritis)	MSS (enthesitis, pain in the sacroiliac joints and spine), eyes (uveitis), CVS (aortitis), RS (ILD, restrictive lung disease)
Laboratory data and pathological findings	ANA, anti-topoisomerase I, anti-centromere and others). Excess matrix deposition, fibrosis, epithelial and endothelial cells dysfunction and death	ANA, anti-Ro/SSA, anti-La/SSB, rheumatoid factor. B-cell and T-cell infiltration	Rheumatoid factor and anti-CCP. Swollen synovium, presence of fibroblast-like and macrophage-like synoviocytes, macrophages and B-cells	ANA, anti-dsDNA, anti-Smith, anti-Ro/SSA, anti-La/SSB, anti-histone (most commonly in drug induced SLE), antiphospholipid antibodies, hypocomplementemia, anemia, lymphocytopenia immune complex formation and deposition, producing inflammation and vasculitis	Serum muscle enzymes (such as creatine kinase and aldolase), an ANA test, anti-Mi-2 antibodies, MDA-5 antibodies and anti-Jo-1 antibody. Capillary abnormalities and loss, perimysial abnormalities, and peri fascicular atrophy	ANA and anti-U1 ribonucleoprotein (RNP). Activation of B-and T-cell, autoantigen modifications	ANCA. ANCA staining pattern on neutrophils and monocytes; patchy infiltrates, vessel wall granulomas, fibrous tissue	HLA-B27, acute inflammatory markers (CRP and ESR) Bone mineralization, capsular fibrosis
Pulmonary involvement	NSIP is the most common followed by OP, DAD/AIP, UIP, and DAH	NSIP and LIP are the most common followed by OP, DAD/AIP and UIP	UIP is the most common followed by NSIP, OP, DAD/AIP, LIP and DAH	NSIP, DAD/AIP, and DAH are the most common followed by OP, LIP and UIP	NSIP and OP are the most common followed by DAD/AIP, UIP and DAH	NSIP is the most common followed by OP, DAD/AIP, UIP, and DAH	UIP is the most common followed by NSIP and OP	Bilateral apical fibrosis
Treatment of pulmonary involvement	CYC and MMF. Autologous stem cell transplantation	Rituximab, antifibrotic drug nintedanib (for gradual fibrosing phenotype)	CS DMARDs (e.g., methotrexate)	High-dose CS in alongside CYC and rituximab in severe cases	CS (mainstay). MMF, AZA and IVIG may be used conjugation.	CS monotherapy or a combination of CS and CYC	CS and CYC or rituximab	Treat secondary infections. Anti-TNF therapy may impact respiratory symptoms

AIP, acute interstitial pneumonia; ANA, antinuclear antibody; ANCA, anti-neutrophil cytoplasmic antibodies; AZA, azathioprine; CNS, central nervous system; CS, corticosteroid; CVS, cardiovascular system; CYC, cyclophosphamide; DAD, diffuse alveolar damage; DAH, diffuse alveolar hemorrhage; DMARDs, disease-modifying antirheumatic drugs; GIS, gastrointestinal system; HF, heart failure; HLA, human leukocyte antigen ILD, interstitial lung disease; IVIG, intravenous immunoglobulin; LIP, lymphoid interstitial pneumonia; MCTD, mixed connective tissue disease; MDA-5, melanoma differentiation-associated gene 5; MMF, mycophenolate mofetil; MSS, musculoskeletal system; NSIP, non-specific interstitial pneumonia; OP, organizing pneumonia; PAH, pulmonary arterial hypertension; RS, respiratory system; TNF, tumor necrosis factor; UIP, usual interstitial pneumonia.

### 2.1. ILD in systemic sclerosis (SSc-ILD)

Systemic sclerosis (SSc) is a chronic systemic immune disease characterized by fibrosis of the skin, lungs, and other vital organs. SSc is characterized by persistent collagen overproduction and connective tissue deposition in various organs. The exact etiology is unknown but involves genetic, environmental, and autoimmune factors. SSc is classified as a class II Human Leukocyte Antigen (HLA) disease, and the HLA genes strongly influence SSc pre-disposition (19, 20). Humoral and cellular immunity alterations associated with specific autoantibodies are a feature of SSc and occur very early in the disease process.

The initial phase in the pathophysiology of SSc ILD is thought to originate in response to a chronic insult to the alveolar epithelial and endothelial cells caused by local inflammation or an environmental stimulus (21). This injury stimulates the recruitment of several inflammatory cells, which infiltrate the alveolar spaces and activate existing immune cells in the lungs (22, 23). Evidence of immune cell activation has been validated in studies demonstrating that CCL2 and other chemokines are released after the epithelial cell injury and influence inflammation and the migration of leukocytes, particularly the activation of M1 (pro-inflammatory) and M2 (profibrotic) macrophages (24). SP-D and KL-6 are glycoproteins that are released from injured epithelial cells and have been found to be elevated in the serum of SSc patients with ILD as opposed to those without ILD supporting the premise of chronic epithelial cell injury (22), further emphasizing the essential role of epithelial cell injury in the SSc-ILD (25).

Adaptive immune system activation leads to the infiltration of T- and B-cells, inducing profibrotic factors and the stimulation of transforming growth factor beta (TGF-β) from its latent state (21), the latter being the principal controller of the immune response (26).

In the absence of effective tissue repair, the resident lung fibroblasts differentiate into myofibroblasts culminating in parenchymal fibrosis. Other potential sources of the myofibroblast include circulating fibrocytes derived from the bone marrow and a biochemical process called the epithelial-mesenchymal transition (EMT) (27, 28). In EMT, alveolar epithelial cells’ polarity is lost, and they lay down an increased extracellular matrix and acquire mesenchymal characteristics (28). However, this theory is not yet proven and has been challenged by more recent research (29).

#### 2.1.1. Clinical manifestations

Patients often present with Raynaud’s phenomenon and may also present with swollen fingers, sclerodactyly (tightening and thickening of the skin), arthralgia or arthritis, malaise, gastrointestinal, renal, cardiac, or pulmonary symptoms (30). The disease can be limited, formerly known as CREST syndrome, or diffuse. Antinuclear antibodies (ANA) are detected in most SSc patients (19, 31). Patients who lack detectable ANA tend to be males and have a reduced frequency of vasculopathy manifestations (32). Several specific antinuclear antibodies are present in systemic sclerosis, including the anti-topoisomerase I (ATA; anti-Scl70), the anti-centromere (ACA), and the anti-RNA polymerase antibodies. These antibodies act as major disease markers, are mutually exclusive and define clinical presentation (31).

Interstitial lung disease is a serious organ involvement in SSc patients and is the principal cause of death (33). Pulmonary involvement occurs in 80% of SSc (34). Traditionally, ILD is linked with diffuse SSc compared to the limited form. However, it is thought that the presence of ATA carries the highest risk of ILD regardless of the cutaneous form (35). Inflammation and fibrosis findings on radiological imaging by HRCT and histopathology characterize SSc-ILD. Non-specific interstitial pneumonia (NSIP) is the major histopathologic type of SSc ILD, while the usual interstitial pneumonia (UIP) form occurs in fewer patients. Moreover, NSIP is characterized by mostly inflammatory cellular/lymphocytic infiltrates and minimal damage or fibrosis (36).

#### 2.1.2. Treatment

Effective SSc-ILD treatment remains a challenge despite the development of new therapeutics.

Currently, SSc-ILD is being treated mostly with immunosuppressive medications like cyclophosphamide (CYC), an alkylating agent that cross-links strands of DNA and RNA and inhibits protein synthesis, or mycophenolate mofetil (MMF) an inhibitor of inosine monophosphate dehydrogenase (IMPDH). Furthermore, autologous stem cell transplantation has also been used as a treatment modality (36). Toxicity remains a major issue in these treatment modalities, although MMF was more tolerable and linked to reduced toxicity (37, 38).

Patients with SSc manifest a variety of B-cell abnormalities, among which is persistent memory B-cell hyperreactivity that may be attributed to overexpression of CD19. This theory is supported by the observation that SSc patients express 20% more CD19 than the normal controls as measured by flow cytometry examination (39). Goswami et al. (40) published a systematic review and meta-analysis in 2021 on using rituximab, an anti-CD20 monoclonal antibody, in treating SSc-ILD that comprised a total of 20 trials. Researchers demonstrated that Rituximab improved pulmonary function results after 6 and 12 months and may be an alternative to MMF and CYC.

The IL-6 receptor monoclonal antibody tocilizumab was licensed by the food and drug administration (FDA) in 2021 to treat SSc-ILD. It was shown to have a persistent favorable effect in the extension phase of the focuSSced trial (41).

Janus kinases (JAK) are a family of cytoplasmic tyrosine kinases occurring in diverse tissues and cells that interact with the Signal Transducers and Activators of Transcription (STATs) transcription factor family (42). When activated by cytokines or growth factors, these transcription factors lead to the differentiation of immune cells and regulate macrophage polarization and the production of a variety of cytokines. JAK inhibitors interfere with the JAK/STAT pathway and halt genetic transcription (43). JAK inhibitors simultaneously exert anti-inflammatory and antifibrotic effects (24) and are promising therapeutic options for SSc-ILD with a favorable outcome, as reported in a recent non-systematic review by Fiorentini et al. (23).

Nintedanib is a triple kinase inhibitor that substantially inhibits the receptors for the fibroblast, vascular endothelial, and platelet-derived growth factors (25). After initial approval and wide-scale use in treating idiopathic pulmonary fibrosis, nintedanib was approved by the FDA in 2019 in SSc-ILD based on the positive results of the SENSCIS study (44). Based on the Phase III trials, the level of evidence supporting nintedanib was high (44, 45) or moderate (46), indicating high confidence in the conclusion that nintedanib significantly slows the annual decline in forced vital capacity (FVC) in SSc-ILD or progressive fibrosing ILD including SSc-ILD. In addition, it has been demonstrated that nintedanib, both by itself and in conjunction with MMF, can slow the deterioration of pulmonary function (47).

Several clinical trials are looking into potential future SSc-ILD therapies. Phase II research examining MMF in combination with pirfenidone is being conducted in the SLS III trial (NCT03221257). MMF with or without the proteasome inhibitor bortezomib is being investigated in another phase II placebo-controlled trial of MMF combination therapy (NCT02370693) (47).

### 2.2. ILD in Sjögren syndrome

SS is an autoimmune disease with sicca symptoms as a hallmark feature due to exocrine gland B-and T-cell infiltration along with focal lymphocytic sialadenitis leading to tissue fibrosis and glandular dysfunction. Similar to other CTD-ILDs, the pathogenesis of SS-ILD involves injury to the alveolar epithelium with impaired healing, which causes fibroblasts to be activated and produce an increased extracellular matrix, resulting in an imbalance between collagen formation and degradation and alveolar fibrosis due to collagen accumulation (48).

Autoreactive effector T-cells originate and reinforce pathogenic B-cell responses. Interleukin 2 (IL-2) is the key stimulator of CD4+ T-cells and controls regulatory T-cells (Tregs) (49). The function of Treg cells was observed to be disrupted in patients with primary SS (50).

Low-dose IL-2 therapy significantly improved disease activity scores and immunological effects in 60 primary SS patients (49). However, the potential role of this therapy on ILD in SS is not well-defined.

In the presence of cytokines, notably IL-21, B-cells get activated through B-cell receptor (BCR) activation and CD40 ligation. IL-21 is a key cytokine that influences B-cell function and is found in higher levels in Sjögren patients than in healthy subjects (51).

Type I IFN system is hyperactivated in primary SS, leading to overproduction of the B-cell–activating factor of the TNF family (BAFF), also known as B Lymphocyte Stimulator (BLyS). BAFF is a potent B-cell activator and plays a crucial function in B-cell proliferation, and differentiation and augments the B-cell migration mediated by CXCL13 (52). It has been suggested that BAFF may be a more useful marker for discovering individuals with high disease activity in the early stages of SS (53).

#### 2.2.1. Clinical manifestations

SS presents with sicca symptoms as a hallmark feature due to exocrine glandular dysfunction (54). Patients can present with dry, itchy eyes, dry mouth with accelerated teeth decay, parotid gland swelling, nasal dryness, chronic dry cough, and dry skin (55). Some patients can have systemic extra-glandular manifestations such as joints, kidneys, nervous system, skin vasculitis, and pulmonary manifestations (56). SS may develop as a primary condition or a result of another connective tissue disorder. Lung involvement in SS manifests mostly as interstitial pneumonitis and dryness of airway mucosal surfaces, but the disease can also result in pleurisy, pulmonary arterial hypertension, pulmonary lymphoma, and amyloidosis (57). SS is distinguished by a range of autoantibodies, specifically anti-Ro/SSA, which is part of the most recent 2016 ACR/EULAR criteria for the classification of Sjögren syndrome (58). ANA, rheumatoid factor (RF), and anti-La/SSB can also be found in SS, but up to 18% of the patients are seronegative (59). Autoantibodies can predate the disease’s clinical manifestation by up to 18–20 years (60).

Interstitial lung diseases can be seen in 20% of SS patients (61). However, the prevalence rises when patients are systematically screened for it regardless of respiratory symptoms. In one report, up to 50% of SS patients had high-resolution computed tomography abnormalities (62). SS patients with ILD were shown to have a lower quality of life and worse prognosis than those without ILD (63).

The most prevalent ILD pattern in individuals with primary SS is NSIP. Less frequent patterns include usual interstitial pneumonia, lymphocytic interstitial pneumonia, and organizing pneumonia, and some patients exhibit a combination of these patterns (64). Patients with advanced age, male gender, or smoking and those with anti-Ro52 antibodies were found to be more likely to be affected by ILD (65, 66).

#### 2.2.2. Treatment

Unfortunately, the only treatments currently available for SS are symptomatic and primarily experimental. Rituximab is a promising treatment for SS-ILD, with small studies showing improvement in clinical symptoms and stabilization of radiological changes for patients treated with rituximab (67, 68).

Belimumab is a BLyS (BAFF) antibody and is an approved treatment for systemic lupus erythematosus. A systematic review on the use of belimumab in autoimmune diseases (69) suggested that the drug has a potential role in SS in general. Still, the study did not address ILD in particular, and no randomized trials were available to include in the review.

Patients with SS can manifest significant BAL lymphocytosis, but this was not proven to be an early sign of severe lung fibrosis (70). Type II pneumocytes in patients with ILD produce a mucin-like glycoprotein called KL-6 (71). The serum level of KL-6 correlates significantly with ILD prognosis and disease severity as defined by HRCT and is considered a potentially useful diagnostic marker for detecting asymptomatic SS-ILD patients (71–73).

Some patients with SS-ILD develop a gradual fibrosing phenotype associated with increased mortality and reduced quality of life. Such patients are eligible for the targeted antifibrotic drug nintedanib (74).

### 2.3. ILD in rheumatoid arthritis (RA)

Rheumatoid arthritis is an autoimmune disease that is characterized by inflammatory arthritis. The arthritis is often symmetrical, and if left untreated, usually results in joint damage from the erosion of bone and cartilage, which results in abnormalities of the joints (75). Numerous cell types are involved, including T-cells, B-cells, macrophages, and fibrocyte-like and macrophage-like synoviocytes. Extra-articular involvement, including lung pathology in the form of ILD, is a well-established complication of the disease (76). There are many identified risk factors for ILD and RA; smoking is one common overlapping risk factor (76). One hypothesis is that rheumatoid arthritis autoimmunity can be generated at non-articular sites, including the lung. Rheumatoid factor (RF) and anti–cyclic citrullinated peptide (anti-CCP) antibody are found in smokers without evidence of rheumatoid arthritis joint involvement. This is important because smoking is the only known modifiable risk factor for RA-ILD. Other studies looking at non-articular sites include gut dysbiosis as a possible mechanism for the development of RA (77). The commonality between these two hypotheses is that these mucosal sites decrease self-tolerance to citrullinated autoantigens.

Numerous noxious substances, including smoke particles, silica particles, textile dust, and bacteria, can harm the lungs and produce immunological reactions linked to RA when combined with susceptibility genes. Dendritic cells (DCs), macrophages, and B-cells are stimulated by toll-like receptor (TLR) stimuli from tobacco smoke, silica dust, textile dust, or from elements of an aberrant microbial flora inside the lungs (78–80). Peptidyl-arginine deiminase (PAD) activation and local extracellular citrullination may result from this mechanism, which could cause the emergence of neo-antigens linked to RA. Furthermore, another effect could be the differentiation of B-cells, which can result in the initial somatic mutation of the immunoglobulin genes and allow B-cells to bind and deliver post-translationally changed autoantigens to T-cells (81). Early in the development of RA-related immune responses and disease, the lungs produce structures resembling germinal centers called induced bronchus-associated lymphoid tissue (iBALT). A local generation of antibodies to citrullinated protein antigens (ACPAs) is encouraged in the lungs by T-cell-dependent B-cell activation. T-cell receptor; APC, antigen-presenting cell (82–84).

#### 2.3.1. Clinical manifestations

Rheumatoid arthritis commonly presents with stiffness and swelling in the hands and feet. While joint involvement is the pre-dominant feature used in the ACR/EULAR criteria for RA, RA also affects other body parts, including the lungs and blood vessels (vasculitis), skin (pyoderma, rheumatoid nodules), eyes (scleritis, keratitis). The most common form of lung involvement in rheumatoid arthritis is ILD (85).

Approximately 10% of patients with rheumatoid arthritis will develop clinically significant ILD (85). However, some studies suggest that this number may be higher, and there is radiographic evidence on CT before patients become symptomatic (86). For some patients, ILD precedes the diagnosis of rheumatoid arthritis (87). One study by Hyldgaard et al. (88, 89) found that 14% of patients with RA-ILD had been diagnosed with ILD 1–5 years before their rheumatoid arthritis diagnosis. It is important to identify and treat RA-ILD as early as possible because it is the second most common cause of death in patients with RA after cardiovascular disease (86).

#### 2.3.2. Treatment

According to case series and clinical evidence, systemic glucocorticoids and/or disease-modifying antirheumatic drugs (DMARDs) may be beneficial in some patients with RA-ILD (7). Data to guide the choice of conventional DMARDs such as methotrexate or mycophenolate, or biologic DMARDs such as abatacept, in RA-ILD is still evolving (90). Known for producing a favorable articular response in resistant RA, biologic treatment with rituximab has also been shown to stabilize RA-ILD in the majority of patients in several reports, and even improve lung status in a small patient subset (91). Among those, a report of 700 RA patients treated with rituximab demonstrated stabilization or improvement of lung disease in 30 out of 44 patients with concurrent RA-ILD, with the remaining 14 patients experiencing ILD progression (92). In that study, a radiologic pattern of UIP and more severe ILD prior to rituximab initiation conferred a poorer response to rituximab and an increased risk of ILD deterioration. Concerns have been raised over rituximab-induced ILD and the general side effect profile of rituximab although the data remains limited in RA patients.

The treatment of RA-ILD has proved to be challenging for many clinicians. Adding to this challenge is the concern that the mainstay treatment for RA with DMARDs increases the risk of pulmonary toxicity. Additionally, little evidence suggests that DMARDs are an effective treatment for ILD (91). Thus, the treatment for joint and lung involvement may need to be evaluated separately. There is evidence for using antifibrotic medications such as nintedanib from the INBUILD trial. This trial demonstrated that in patients who received nintedanib, the annual rate of decline in the FVC was significantly lower than in those who received the placebo (46). Additional research and clinical trials are still needed to determine the optimal treatment for RA-ILD.

### 2.4. ILD in systemic lupus erythematosus (SLE)

Systemic lupus erythematosus (SLE) is a multisystem autoimmune disorder of unknown etiology that involves a complex interplay of environmental and genetic factors. Much of the pathology in SLE is believed to stem from failure of self-tolerance, dysregulated apoptosis, formation of autoantibodies, and immune complex formation and deposition, producing inflammation and vasculitis that culminate in the multisystemic manifestations of SLE. Among the systems involved, pulmonary involvement is common in the form of pleuritis and pleural effusions and, to a lesser extent, ILD (93).

The underlying mechanisms of lung involvement in SLE, particularly ILD, also remain poorly defined, and whether it arises from progression of lung inflammation and consequent parenchymal damage and fibrosis or not is also unclear. Lung inflammation has been associated with circulating immune complexes, increased levels of certain cytokines, and neutrophils (94). The initial inflammatory insult damages and activates epithelial and endothelial cells in the lung, with production of mediators that attract various cells including neutrophils. The role of neutrophils in perpetuating the inflammatory response in SLE has been highlighted through the process of NETosis in which released DNA and nuclear antigens, which are primary autoantigens in SLE, further drive inflammation (94). Additionally, the role of certain cytokines in the development of pulmonary pathology has been proposed on the basis of observations demonstrating augmented systemic levels of IFN-gamma, IL-6, and IL-8 in comparison to SLE patients without lung involvement (95, 96). The exact mechanisms that govern progression to fibrosis, however, are poorly understood.

#### 2.4.1. Clinical manifestations

Like other autoimmune disorders, SLE primarily affects women. It has a variety of clinical presentations and is sometimes referred to as the “great mimicker” because of this heterogeneity. Common clinical manifestations include a malar rash, mouth ulcers, hematologic abnormalities, cardiovascular abnormalities, neuropsychiatric abnormalities, synovitis, joint tenderness or stiffness, and renal involvement.

Pulmonary involvement is a common finding in SLE. Although only 5% of patients have pulmonary involvement as their presenting symptom, nearly half of all patients with SLE will develop it in the future (97). There are two major categories of lung conditions in lupus: acute lupus pneumonitis and ILD. Interstitial pneumonitis is a less frequent occurrence in SLE than other CTDs and has received less attention than other CTD-ILDs, particularly SSc-ILD, but it shares similar pathogenic mechanisms (98). The most frequent histopathology types are acute interstitial pneumonia and NSIP, and high-resolution CT of the lung shows ground glass opacities (99, 100).

Patients with ILD secondary to SLE are more likely to present at an earlier age than those with ILD who do not have an autoimmune condition. One study by Hussain et al. (101) found that the average age at ILD diagnosis in a patient with SLE was 59.28 compared to 72.32 years in those without autoimmune disease. The same study also looked at the racial distribution and found that African American populations with ILD, compared to other races, were more likely to have an underlying autoimmune disease, including SLE, mixed connective tissue disease, myositis, or scleroderma. A multicenter study of 513 patients with SLE found that ILD was present in 1% at the time of onset, 4% of patients within the following year, and 8% by 12 years. ILD seemed to increase with the duration of disease and also with age (102, 103).

Immunologic criteria for SLE include antinuclear antibody (ANA) and SLE-specific antibodies: anti-dsDNA and anti-Smith antibody (104, 105). None has shown a good correlation with development of ILD (106). Other antibodies in SLE include anti-histone antibodies (which are associated with drug-induced lupus), anti-Ro/SSA, anti-La/SSB, as well as antiphospholipid antibodies. Additional laboratory testing may show anemia, lymphocytopenia, and hypocomplementemia (107).

Similar to other diffuse CTD-ILDs, the most typical symptoms include non-productive coughing, pleuritic chest discomfort, and persistent exertional dyspnea. Physical examination may reveal bibasilar crackles, cyanosis, and fever. As opposed to idiopathic ILD, clubbing is less frequent in SLE.

Biopsies collected from people with ILD related to SLE have been shown in histological examinations to have lymphocytic, mononuclear interstitial, and peribronchiolar infiltrates (108). A study done by Chen et al. (109) found that among Chinese patients with SLE, there were several statistical differences compared to those without ILD: including older age, increased illness duration, lower levels of anti-dsDNA, high C3 levels, increased ratios of Raynaud’s phenomenon, moist rales and tachypnea. This poses the question of whether there should be increased screening for ILD in patients with these biomarkers or with Raynauds.

#### 2.4.2. Treatment

Treatment of SLE-ILD is primarily based on case reports, case series, or expert opinion and there are varying degrees of consensus on treatment since there are few randomized controlled studies to support the current treatments. Current therapies frequently involve high-dose corticosteroids in conjunction with medications like cyclophosphamide and rituximab in severe instances (100, 110). In milder cases or to preserve long-term disease control, steroid-sparing medications like MMF and azathioprine may be utilized (111, 112). Belimumab is an approved treatment for SLE (69), but data on its utility for SLE-ILD is limited, with few case reports of its favorable effect (113). In addition, as with other autoimmune-related ILD, those patients who develop fibrosing phenotype are eligible for treatment with the targeted antifibrotic drug nintedanib (74).

### 2.5. ILD in dermatomyositis and polymyositis

Dermatomyositis (DM) and polymyositis (PM), which are categorized under idiopathic inflammatory myopathies, are immune-mediated myopathies characterized by the involvement of the muscles as well as extramuscular symptoms (114). The exact pathogenic mechanisms responsible for DM are still unknown. Application of genomic technologies to DM skin and muscle samples has led to the hypothesis that DM pathology is caused by exposure to a type 1 interferon (IFN) that damages keratinocytes, myofibers, and capillaries (115–117). The microscopic pathology of DM, which includes perifascicular atrophy, capillary abnormalities and loss, and perimysial abnormalities, is distinct from all other muscle illnesses. Perifascicular myofibers, which are situated at the borders between fascicles and perimysial connective tissue, are preferentially harmed. These myofibers are atrophic and exhibit neonatal myosin (MHY8), vimentin, and natural cell adhesion molecule (NCAM), which are often linked to regeneration (118, 119). However, it seems improbable that these myofibers are regenerating. Moreover, PM is thought of as a condition in which myofibers are invaded by lymphocytes, particularly CD8+ cytotoxic T-cells. A protein called melanoma differentiation-associated gene 5 (MDA-5) serves as an intracellular pattern-recognition receptor that can identify danger signals in double-stranded RNA. Type I interferons are produced in considerable quantities after MDA-5 is activated the reason behind this remains unknown (120). It is hypothesized that this leads to the activation of non-inflammatory macrophages which produce IL-10 and TGF-β and are involved in the progression of lung fibrosis (121, 122).

#### 2.5.1. Clinical manifestations

Both DM and PM have a wide range of clinical symptoms and are multisystem diseases. The majority of individuals show symptoms of muscular inflammation and proximal skeletal muscle weakening (118, 123). DM is characterized by a number of distinctive skin eruptions, such as Gottron papules and the heliotrope eruption (124). Patients suffering from myositis-ILD commonly experience shortness of breath and non-productive cough. However, some patients may not show any symptoms and ILD is only discovered through an abnormal lung examination or chest X-ray (125). ILD occurs in 20–80% of cases with DM and PM patients and is a common cause of death among them (126, 127). DM and PM have been linked to a number of ILD histologic patterns with NSIP and OP being the most common pattern (128). A complete blood count and differential, hepatic and renal function assessments, and a brain natriuretic peptide (BNP) or N-terminal-proBNP test are frequently included in initial laboratory evaluations. Additionally, serum muscle enzymes (such as creatine kinase and aldolase), an antinuclear antibody test, anti-Mi-2, and an anti-Jo-1 antibody are done if the diagnosis of DM or PM has not yet been established (129). The clinical examination and outcomes of the preliminary tests serve as the basis for further testing (e.g., further antisynthetase antibodies, MDA-5 antibodies which are associated with rapidly progressive ILD, and testing for an overlap syndrome) (128, 130, 131). The diagnosis of ILD in patients with a history of DM and PM can typically be confirmed through a combination of their symptoms, chest imaging (e.g., HRCT), and pulmonary function tests (132). It is recommended that all individuals diagnosed with myositis, regardless of their respiratory condition, undergo complete pulmonary function tests (PFTs) at the time of diagnosis and then annually afterward (127). Furthermore, a lung biopsy is not typically needed. In cases where the symptoms or imaging appear atypical (for example, accompanied by a fever, rapidly worsening, or occurring during immunosuppressive treatment), bronchoscopy with BAL may be performed to rule out an infection or drug-related cause (133). If the diagnosis is still uncertain, a lung biopsy may be necessary.

Karampitsakos et al. (128) reported that ILD is a common feature of inflammatory myopathies, including those without muscle involvement, and may occur before the onset of muscle symptoms. Consequently, physicians should be aware that cases of organizing pneumonia without obvious muscle involvement may be related to myopathy. When patients with bilateral organizing pneumonia do not respond to antibiotics, physicians should thoroughly evaluate them for myositis-associated ILD, especially if they have certain autoimmune markers like anti-MDA5, which can have a negative impact on prognosis (128).

#### 2.5.2. Treatment

Diagnosing and treating myositis-ILD presents challenges that are best addressed through a collaborative effort between experienced rheumatologists and pulmonologists (134). There are currently no standard guidelines for managing patients with myositis-ILD. Treatment plans are based on the expertise of medical professionals and can vary between medical centers. Corticosteroids (CS) are used as a baseline therapy for all patients with myositis-ILD due to their rapid onset of action and availability. The starting dose is determined based on the severity of the patient’s disease and any underlying health conditions, such as diabetes or osteoporosis (127, 135). Because of the potential long-term consequences of taking prednisone and the risk of disease flare-ups, a steroid-sparing agent, such as MMF or AZA, is usually prescribed concurrently with prednisone to allow for a gradual reduction in prednisone use (127, 136). Although there is no clear evidence supporting the use of one steroid-sparing agent over the other, MMF is preferred due to a lower risk of gastrointestinal symptoms and lab abnormalities. IVIG is used as adjunctive therapy for patients with refractory disease, severe skin involvement, or severe myositis (127). Regarding tacrolimus, it is used only for patients who do not show improvement or continue to have declining pulmonary function after 3 months of treatment with steroids and an anti-metabolite (MMF or AZA). In cases of severe combined ILD and myositis, tacrolimus may be chosen as a first-line steroid-sparing agent. However, when possible, MMF or AZA is favored due to the potential for long-term side effects and the need for monitoring drug levels in patients taking tacrolimus. Rituximab is also used as an additional therapy for patients with persistent or progressive disease despite treatment with prednisone and an anti-metabolite or calcineurin inhibitor. CYC is rarely used due to concerns about side effects and lack of evidence that it is more effective than other immunosuppressants for ILD. Despite treatment with immunomodulatory therapy, some patients with myositis-ILD may develop a progressive fibrotic form of ILD. In cases where there is radiographic evidence of worsening bronchiectasis or the development of honeycomb changes, anti-fibrotic therapy with nintedanib or pirfenidone is added (127).

### 2.6. ILD in mixed connective tissue disease (MCTD)

Mixed connective tissue disease is a rare autoimmune disorder affecting multiple body systems, including the joints, skin, lungs, blood vessels, and muscles. It is characterized by the presence of antibodies to a protein called ribonucleoprotein (RNP) and symptoms of overlap between SLE, scleroderma, and polymyositis (137, 138). The exact cause of MCTD is unknown, but it is believed to be a combination of genetic and environmental factors. It is widely known that autoimmunity plays a part in ILD linked to CTD. Increased levels of proinflammatory and profibrotic mediators have been linked to the development of ILD and might play an important role in MCTD-ILD. These mediators include lipids, prostanoids, growth factors, chemokines, and cytokines (99). In MCTD-ILD, the immune complex complements C3 factor, anti-U1-RNP, and CH50 are all substantially expressed. Gunnarsson et al. (139) reported that anti-Ro52 antibodies are associated with MCTD-ILD.

#### 2.6.1. Clinical manifestations

Mixed connective tissue disease might present with non-specific early clinical symptoms including general malaise, arthralgias, myalgias, and low-grade fever (140). Raynaud phenomenon and the absence of severe renal and central nervous system disease help differentiate MCTD from SSc and SLE (141–143). Dyspnea, dry cough, and pleuritic chest pain are early signs of pulmonary involvement in MCTD that should be taken seriously (144). One of the most serious complications of MCTD is ILD. ILD is thought to be caused by the immune system attacking the lung tissue. ILD can occur in about up to 50% of patients with MCTD. Furthermore, a sensitive diagnostic test to identify ILD is HRCT. Septal thickening, ground-glass opacities, non-septal linear opacities, and peripheral or lower lobe pre-dominance are the most prevalent HRCT results and are most comparable to the SSc findings (145, 146).

#### 2.6.2. Treatment

As there have been no randomized clinical trials to help direct treatment, it is believed that MCTD is an incurable condition. The treatment is mostly determined by the proven effectiveness of certain medications for conditions that are similar to SLE, scleroderma, or polymyositis. In the early inflammatory stage of ILD associated with MCTD, CS monotherapy or a combination of CS and CYP may be beneficial in stopping the progression of ILD and the development of fibrosis (147).

### 2.7. ILD in antineutrophil cytoplasmic antibody (ANCA)-associated vasculitis

Antineutrophil cytoplasmic antibody-associated vasculitis (AAV) is a systemic vasculitis that mainly affects small blood vessels. It consists of three different clinical syndromes: microscopic polyangiitis (MPA), granulomatosis with polyangiitis (GPA), and eosinophilic granulomatosis with polyangiitis (EGPA) (148). Association between ANCA-associated vasculitis (AAV) and interstitial lung disease has been increasingly reported over the past 3 decades (148). The significant pre-dominance of myeloperoxidase (MPO) or (P-ANCA) related AAV-ILD was reported compared to other AAV subtypes (149). The prevalence rate of ILD was reported in up to 45% of MPA patients and 23% of GPA patients (148). Mucin 5B (MUC5B) promotor has a role in airway clearance and host defense against bacteria, with one of its variants rs35705950T identified (148) and was found to increase the risk of IPF in AAV-ILD patients in a case-control study from Japan (148, 150). The impact of ANCA is based on the activation status of neutrophils. Once primed, neutrophils can be bound by ANCA to relevant antigens on the cell membrane. This leads to abnormal activation through crosslinking of MPO or PR3 or by binding to Fc receptors. ANCA’s binding to neutrophils can increase the interaction between neutrophils and endothelial cells, causing microvascular damage, which supports ANCA’s potential role in causing systemic vasculitis (151, 152).

The exact cause of ILD in AAV is not well understood. However, Kagiyama et al. (153) postulated that repeated episodes of alveolar hemorrhage due to pulmonary capillaritis may lead to the development of pulmonary fibrosis. Also, Guilpain et al. (154) suggested that oxidative stress, particularly the production of hypochlorous acid (HOCl) through the interaction of MPO with anti-MPO antibodies, could drive the fibrotic process observed in MPA. Pathology of ILD associated with ANCA-positive vasculitis was evaluated in a few studies. In a case series study of 9 patients with MPO-ANCA idiopathic interstitial pneumonia (IIP), small airway disease was found in all the patients, lymphoid follicles in seven patients, UIP with areas of NSIP in eight patients, UIP and diffuse alveolar damage (DAD in two patients and no vasculitis in any patients (155).

In another study of 18 patients evaluated with surgical lung biopsy, 65% were noted to have UIP patterns and 48% with UIP and other atypical findings of UIP related to IPF (148, 156).

#### 2.7.1. Clinical manifestations

Individuals who have both AVV usually exhibit general symptoms such as fever, feeling unwell, loss of appetite, weight loss, muscle aches, and joint pain. These early warning signs can persist for several weeks to months without showing specific signs of organ damage. Later, specific organ problems may become evident such as the Vascular system (wall thickening), skin (purpura), nervous system (mononeuritis), and renal system (glomerulonephritis) (157).

Pulmonary involvement in AVV can manifest as pulmonary nodules/consolidation, more common with GPA, diffuse alveolar hemorrhage that is often associated with renal vasculitis, tracheobronchial stenosis, fibrosing interstitial disease, and IPF (158). Although the median age at onset of MPA vasculitis is 55 years, MPA-associated ILD usually presents in patients 65 years and older, with a higher incidence in males (148).

In a retrospective study on 745 north American patients with IPF, 4.0% of the patients were positive for ANCAs at the time of diagnosis and 25–33% of them were diagnosed with vasculitis during a median duration of follow-up 18 months (148, 159). However, isolated IPF cases with positive ANCA in the absence of any AAV manifestations (148, 160).

Interstitial lung disease in AAV, especially anti-MPO ILD with or without systemic manifestations, is becoming more recognized, ANCA is not typically included in ILD workup and standardized criteria for diagnosis and classification of autoimmune-ILD are not available. More studies are needed to evaluate the association between ANCA positivity and ILD, its impact on disease course and prognosis, and potentially beneficial treatment options.

In a multicenter study in Japan that included 150 patients with MPA, 66% had interstitial lung abnormality (148, 161). The most common radiologic pattern with ANCA/AAV-related ILD is UIP (78%), NSIP (13–64%), less common patterns include organizing pneumonia and desquamative interstitial pneumonia (148, 161, 162). Other described radiologic patterns include ground glass opacities (GGO), honeycombing, nodular pattern, consolidation, reticulation, and interlobular septal thickening (148, 156, 163, 164).

#### 2.7.2. Treatment

There are numerous studies about ANCA-associated systemic vasculitis, but few studies address ANCA-associated ILD, with less defined treatment for ANCA-associated ILD in the absence of systemic vasculitis (148). Retrospective case reports and limited data, especially with non-UIP patterns revealed an improvement in PFT in patients treated with immunosuppressive agents (148, 165). However, a study on 62 patients showed no improvement in the prognosis with immunosuppressive treatment (166). A study on 49 patients who were treated with glucocorticoids and cyclophosphamide or rituximab as induction therapy showed higher 1- and 5-year survival rates compared to those who were treated with glucocorticoids alone (148, 164).

As with all other CTD-ILD, the INBUILD study suggested a benefit of nintedanib in the progressive fibrosis-ILD (PF-ILD) subtypes (148, 167). Moreover, antifibrotic rather than anti-inflammatory therapy should be prioritized in patients with a high risk for infection, a UIP phenotype and no systemic vasculitis features (167).

### 2.8. Ankylosing spondylitis (AS)-associated lung disease

Ankylosing spondylitis (AS) is a chronic inflammatory disease that is part of the broader entity termed axial spondyloarthropathies. It is pre-dominantly a disease of the axial skeleton, including the sacroiliac joints and spine, and less commonly, peripheral joints (168). Extra-articular involvement may occur and includes ocular (e.g., uveitis), cardiovascular (e.g., aortitis), and pulmonary manifestations (e.g., ILD) (169–171). In 85–90% of AS cases, there is an association with HLA-B27, an MHC variant presumed to contribute to disease pathogenesis *via* aberrant presentation of unidentified antigens that trigger inflammation in the axial joints (172). Cytokines such as TNF-alpha, IL-17, and IL-23 are important mediators of the disease and serve as therapeutic targets for AS (173, 174).

Pulmonary disease in AS primarily follows a restrictive pattern of changes that is largely attributed to musculoskeletal disease, and to a lesser extent, parenchymal lung involvement (171, 175). Spinal and costovertebral joint fusion and rigidity, enthesitis in the anterior chest wall and the ensuing decline in chest wall mobility restrict chest wall expansion (171). Other pulmonary changes, including apical fibrosis, have no clear pathophysiology but are possibly related to altered mechanical stresses and diminished ventilation in the apices of the lung, chronic inflammation, and recurrent aspiration pneumonitis secondary to esophageal dysfunction (171).

#### 2.8.1. Clinical manifestations

The characteristic presenting symptom of AS is insidious-onset inflammatory back pain in an adult below the age of 40 that may or may not be associated with peripheral articular and extra-articular symptoms at presentation. Testing for suspected AS will include conventional radiography of the sacroiliac joints, revealing sclerotic to erosive changes as well as joint space narrowing indicative of sacroiliitis, in addition to a spine X-ray, revealing loss of lumbar lordosis, bony growths (i.e., syndesmophytes) and a bamboo spine in more advanced stages of the disease. More advanced imaging with CT and MRI proves useful in cases of equivocal sacroiliac X-ray findings with clinical suspicion of AS. Laboratory testing will likely reveal HLA-B27 positivity, along with elevation of non-specific inflammatory markers such as CRP and ESR (169, 176).

Pulmonary disease in ankylosing spondylitis is a rare and late occurrence, although onset has been reported to vary but occurs at an average of 15 years following the onset of articular symptoms (171, 177, 178). Pulmonary disease in AS follows an insidious course. Among the most common pulmonary changes observed in AS is a restrictive pattern demonstrated on pulmonary function tests, including mildly to moderately diminished vital capacity. This can be due to isolated musculoskeletal disease or concurrent parenchymal lung pathology which can occur in the form of ILD, apical fibrosis, emphysema and bronchiectasis (179).

In 1.3–15% of patients, plain radiography may demonstrate apical fibrosis but is often an asymptomatic finding (175, 180, 181). Nevertheless, colonization and superinfection of apical cysts or cavities may occur, in which Aspergillus fumigatus is the most common culprit, followed by mycobacteria, and may produce symptoms of fever, hemoptysis and dysnpea. Additionally, apical fibrobullous disease may pre-dispose to spontaneous pneumothorax (171). HRCT better reveals parenchymal lung changes as well as pleural thickening, cavitation and bronchiectasis (175). Histopathologic lung specimens of AS patients with pulmonary involvement may reveal patchy pneumonia, scarring, bullae formation, bronchial dilatation, bronchiolitis obliterans or organizing pneumonia (177, 178, 182–184).

#### 2.8.2. Treatment

The main way to manage respiratory issues related to ankylosing spondylitis is by treating any secondary infections with either systemic or locally administered antibacterial or antifungal agents (171). Anti-TNF-α therapy has been proven effective both clinically and in imaging studies for treating ankylosing spondylitis and is an option for patients who are unresponsive to non-steroidal anti-inflammatory drugs (185). Although drugs with anti-inflammatory properties, such as infliximab, etanercept, and adalimumab, are utilized to treat ankylosing spondylitis, it remains uncertain how they impact respiratory symptoms (171, 185, 186). Also, there is no evidence that any form of therapy can change the progression of apical fibrobullous disease.

## 3. Exposure-related lung diseases

Most of the diseases listed in this section are discussed briefly.

### 3.1. Post-infectious lung diseases

Post-infectious immune-mediated ILD is a condition that arises when infectious agents stimulate an immune response that attacks the lungs or when the healing process becomes abnormal following an infection (187). Post-infectious ILD has been described following various infectious agents, and interest has grown dramatically with the advent of coronavirus disease 2019 (COVID-19).

Infections may contribute to the development of ILD (187). It is postulated that the pathogenesis of infection-induced ILD involves either direct epithelial injury followed by an aberrant healing process or activation of the immune system and the subsequent generation of pro-fibrotic and pro-inflammatory cytokines, both of which may cause pulmonary fibrosis (188).

Viral infections and their association with ILDs have received significant attention in recent years, particularly during the Coronavirus disease 2019 (COVID-19) pandemic, despite the fact that the mechanisms behind virus-induced ILDs remain unknown. Alterations of fibrosis-related pathways and mechanisms, such as decreased expression of angiotensin-converting enzyme 2 (ACE2), overexpression of the TGF-β/Smad pathway, increased production of pro-fibrotic and pro-inflammatory cytokines, as well as increased stress in the endoplasmic reticulum, have been shown to contribute to epithelial-mesenchymal transformation, a major mechanism of pulmonary fibrosis (188).

Infection with influenza may cause lung fibrosis by activating the TGF-β/Smad pathway, which can trigger the overexpression of pro-fibrotic gene. Jolly et al. (189) reported in a murine study that influenza A infection induced αvβ6 integrin-mediated TGF-β activity in epithelial cells through activation of toll-like receptor 3 (TLR3), leading to the death of epithelial cells and an increase in collagen deposition. Shatskaya et al. (190) discovered, in influenza A-infected mouse models, that an increase in TGF-β expression may affect the balance of activation and inhibition of SMAD proteins, leading to epithelial-mesenchymal transition and progression to fibrosis.

Herpesviruses have been shown to cause pulmonary fibrosis. Malizia et al. (191) found that Epstein–Barr virus (EBV)-infected human alveolar epithelial cells had increased TGF-β1 expression, which was decreased by Ganciclovir treatment. Mora et al. (192) discovered that infection of IFN-γ receptor-deficient mice with Murine Gammaherpesvirus 68 (MHV-68) led to epithelial injury, myofibroblast transformation, enhanced TGF-β expression, and ultimately deposition of interstitial collagen. Cook et al. (193) observed aberrant TNF-alpha expression by polymerase chain reaction (PCR) and a substantial increase in lung fibrosis in murine Cytomegalovirus (CMV) reactivation models 2 weeks after infection, compared to uninfected controls. In a meta-analysis including 1,287 patients, Sheng et al. (194) found that the presence of EBV, CMV, Human betaherpesvirus 7 (HHV-7), and Human Herpesvirus-8 (HHV-8) significantly increased the risk of developing idiopathic pulmonary fibrosis.

Limited information is available about the role of bacteria and fungi in developing ILDs. Molyneaux et al. (195) found that a higher bacterial load in bronchoalveolar lavage was predictive of a decline in lung function and increased mortality in idiopathic pulmonary fibrosis (IPF) patients and that *Haemophilus*, *Streptococcus*, *Neisseria*, and *Veillonella* spp. were more prevalent in IPF patients than in control subjects. In addition, a significant prevalence of *Pneumocystis jirovecii*, a fungus, was seen among immunocompetent ILD patients (196). However, more clinical and translational research is needed to understand the pathogenesis of ILD caused by these infectious agents.

It can be challenging to establish the connection between infection and interstitial lung disease (ILD) in human clinical studies because the infection may exacerbate existing ILD or cause new-onset ILD through other mechanisms. Therefore, many animal studies are needed to help clarify how different infections contribute to developing ILD. As mentioned earlier, there are numerous subtypes of ILD with varying degrees of inflammation and fibrosis. Still, current animal studies have primarily focused on elucidating the pathogenesis of lung fibrotic changes. Further research is needed to examine the immune system pathways involved in the various subtypes of ILD. In the future, when uncontrolled infection is suspected as a possible cause of ILD, infection-induced ILD should be considered as a differential diagnosis to allow for early treatment and improved prognosis.

#### 3.1.1. Clinical manifestations

The clinical presentation of post-infectious immune-mediated ILD can be diverse. It may be either acute or chronic, with the clinical course differing between different pathogens and being unpredictable in some cases (187). Many patients with certain infectious diseases have also had long-term residual radiological abnormalities, such as evidence of inflammatory and persistent fibrotic-like changes visible on chest CT scans (188). The most common pathological finding in post-infectious ILD is the infiltration of immune cells and the development of fibrotic changes, which can manifest as various forms of interstitial pneumonia, including diffuse alveolar damage, pleuroparenchymal fibroelastosis, granulomatous inflammation, or eosinophilic pneumonia upon histopathological examination (187).

### 3.2. Post-COVID-19 ILD

Coronavirus disease 2019 (COVID-19) has been the leading cause of morbidity and mortality since the identification of severe acute respiratory syndrome coronavirus 2 (SARS-CoV-2) in 2019 (197, 198).

Coronaviruses are currently being extensively studied for their role in the underlying pathogenesis of ILDs. Kim et al. (199) infected human dipeptidyl peptidase 4 (hDPP4)-transgenic mice with Middle East Respiratory Syndrome-Coronavirus (MERS-CoV) and identified histological indicators of progressive pulmonary fibrosis, increased production of pro-fibrotic cytokines, and inflammatory response in the lungs.

Characterized by acute respiratory distress syndrome (ARDS) like hyperinflammation and endothelial dysfunction, these post-COVID changes can be summarized as a fibroproliferative response that can persist for a long period. Lung injury in severe COVID-19 disease is associated with an increased risk of progression to ILD. ILD has a broad spectrum combining inflammatory and fibrotic features. It is crucial to understand the primary contributors of ILD in COVID-19, how to mitigate the risk factors involved, and management strategies to approach the long-term consequences.

Fibrotic ILD/pulmonary fibrosis is the most common long-term pulmonary sequela of COVID-19 as suggested by fibrotic features seen on the histopathology of COVID-19 patients (200). Pathogenesis of ILD secondary to COVID-19 is thought to be caused by multiple etiologies, including the development of cytokine storm and abnormal repair mechanisms (201). According to Ravaglia et al. (202), the immune component of underlying pathology is determined by transbronchial lung cryo-biopsy along with CT imaging (203) suggestive of residual lung disease. Different morphological patterns can be observed in three different groups: (1) chronic fibrosis, (2) organizing pneumonia and fibrosing interstitial pneumonia, and (3) diffuse vascular injury within normal parenchyma. Immunophenotypical changes are also seen due to abnormal expression of STAT3 in hyperplastic pneumocytes and PD-L1, IDO, and STAT3 in endothelial cells.

#### 3.2.1. Clinical manifestations

Lungs are the primary target organ of SARS-CoV-2, which manifests as critical pneumonia, often with long-term pulmonary complications ranging from impaired respiratory physiology, and residual shortness of breath to vascular compromise (204).

#### 3.2.2. Treatment

Being a relatively new condition, pharmacological therapies for COVID-19-associated ILD are still being studied. Currently, high-dose corticosteroid (dexamethasone) has been proven to be the first line of management (200, 203). Prior to COVID-19, antifibrotic drugs like pirfenidone were used for IPF (205). Since fibrosis is a key feature of post-COVID disease, such classes of drugs can be explored for the management of COVID-associated ILD. Ntatsoulis et al. (206) suggest therapeutic targeting of Autotaxin (ATX), a lysophospholipase D responsible for extracellular production of lysophosphatidic acid (LPA), a signaling lysophospholipid that effects pulmonary and immune cells. It is shown that COVID-19 increases the production of IL-6 as well as ATX, hence anti-ATX therapy could be an effective treatment for COVID-19. Treatment for prolonged COVID-19 symptoms continues to evolve, and more research should be promoted to further understand underlying etiologies and pathogenesis, which lead toward targeted therapies (207). The role of immunomodulatory or anti-fibrotic therapies in COVID-associated ILD is still questionable. Immunomodulators may potentially promote the reversal of “inflammatory” changes whereas anti-fibrotic (e.g., nintedanib used in progressive-fibrosing ILD) may reduce “fibrotic-like” changes (208).

In a recent retrospective report evaluating post-COVID-19 ILD using machine learning radiographic models, 13 out of 232 patients followed after severe COVID-19 infection developed fibrotic-like changes on HRCT with persistent functional impairment at 6 months of follow-up. After antifibrotic therapy with pirfenidone or nintedniib, improvement was observed on imaging and PFTs in these patients. Interestingly, given the unvaccinated status of the majority of the cohort in their study and in all of the 13 patients with fibrotic-like changes receiving antifibrotic therapy, this raises the possibility that vaccination against COVID-19 might also prevent the sequelae of COVID-19 infection including post-COVID-19 ILD, and further research is needed to substantiate these observations (209).

### 3.3. Drug-induced ILD

When exposure to a drug causes inflammation and eventually fibrosis of the lung interstitium, it is known as drug-induced interstitial lung disease (DIILD) (210). Over 350 drugs may cause DIILD, and patient presentation and imaging vary vastly between drugs and between patients on the same drug. Risk factors for DIILD include age, smoking, sex and any underlying lung condition (210). All available tests for DIILD lack specificity, thus it is a diagnosis of exclusion and is made based on establishing a temporal relationship between symptoms and drug exposure. This poses a significant challenge for treating physicians. A recent systematic review of observational studies was conducted and found that cancer drugs were the leading cause of DIILD, followed by DMARDs, antibiotics, non-steroidal anti-inflammatory agents, psychiatric medications, and anti-arrhythmic agents. In rheumatology, it is especially difficult to analyze DIILD given the background prevalence of ILD and the immunosuppressive nature of DMARDs, and associated risk of infections that often present as respiratory symptoms. The lack of diagnostic biomarkers for DIILD has limited treatment options and drug development. Though there have been several attempts to find an association between treatment with methotrexate, leflunomide, and biologic DMARDs, definitive causation and the underlying mechanism that rheumatic drugs may induce alveolar inflammation, interstitial inflammation, and/or interstitial fibrosis remains unknown (211). DIILD and other ILDs are difficult to distinguish clinically from one another. Also, the same drug may produce varying imaging patterns and vice versa (210).

#### 3.3.1. Treatment

Treatment of DIILD requires empiric drug discontinuation (210). The reported efficacy of glucocorticoid (GC) therapy to treat DIILD varies widely, and its use is generally reserved for patients with rapidly progressive or more severe pulmonary toxicity. Although there is currently no consistent evidence on which to base recommendations for either drug withdrawal or GC use in DIILD, clinical experience and available literature demonstrating improvement or resolution with this treatment modality suggests a link between immunity and ILD.

### 3.4. Hypersensitivity pneumonitis (HP)

Hypersensitivity pneumonitis (HP), also known as extrinsic allergic alveolitis, is a complex syndrome with variable severity, clinical presentation, and natural history. Agricultural dusts, bioaerosols, microbes (fungal, bacterial, or protozoal), and certain reactive chemical species are just a few of the many inciting agents that have been identified. HP can be classified as acute, subacute, or chronic (212, 213). However, guidelines from the American Thoracic Society, Japanese Respiratory Society, and Asociación Latinoamericana de Tórax (ATS/JRS/ALAT) now classify HP into two subtypes: non-fibrotic and fibrotic (214). These subtypes are easier to differentiate and have a stronger correlation with clinical outcomes. Regardless of whether HP is non-fibrotic or fibrotic, common symptoms include difficulty breathing and coughing. During a physical examination, crackles, mid-inspiratory squeaks, and occasionally wheezes may be detected, and clubbing of the fingers may be observed (215). The clinical, radiologic, and histopathologic characteristics of HP overlap with those of other ILDs, making a diagnosis difficult. Additionally, it may not be feasible to pinpoint the exposure that caused the disease. The prognosis and treatment are significantly impacted by the presence of lung fibrosis on HRCT (214, 216). Individuals with HP should undergo routine monitoring to determine whether their condition is progressing. The inciting antigen must be avoided. Although frequently used to treat HP, immunosuppression has not been proven to delay the development of fibrotic disease. For fibrotic HP with a progressive phenotype, the tyrosine kinase inhibitor nintedanib is an approved treatment option (216).

### 3.5. Occupational lung diseases

Occupational lung diseases include pneumoconioses (interstitial lung diseases). Pneumoconioses, accumulation of mineral dust and inorganic dust, followed by lung response, can be classified as fibrogenic (caused by silica, coal, talc, or asbestos), innocuous or inert (caused by iron, tin, or barium), granulomatous (caused by beryllium), or giant cell pneumonia linked to inhalation of hard metals (e.g., cobalt) (217, 218). Previous exposure history and suggestive radiographic findings help in the diagnosis. Tissue biopsy is typically not necessary to confirm the diagnosis when the exposure history and radiographic pattern are characteristic due to the typical radiographic appearance of the most prevalent pneumoconioses. Specific imaging patterns in pneumoconiosis include focal nodules and masses, diffuse lung disease and pleural plaques (219). Occupational asthma is the most frequently diagnosed occupational lung disease. In the past, research on occupational lung diseases has primarily focused on diseases resulting from exposure to hazards relevant to high-income countries and obvious hazardous occupations such as coal mining-induced silicosis. But, in 2019, peer-reviewed publications have expanded the scope to include low- and middle-income countries and previously neglected occupations such as dry cleaning and animal husbandry. Thanks to technological advancements and a better understanding of the causes of the disease, researchers and clinicians can now implement improved risk analysis, screening, and mitigation strategies not only to treat occupational diseases but also to identify at-risk populations and establish measures to prevent or reduce the negative effects of workplace hazards. As occupational lung diseases are increasingly recognized as a global threat in various occupations, research is progressing, leading to the development of better treatments and preventative measures, which promote workers’ rights and ensure their continued good health (220). There is currently no specific cure for most occupational lung diseases. Therefore, a multidisciplinary approach is required for the prevention and control of occupational lung disease progression.

### 3.6. Respiratory bronchiolitis-ILD

Respiratory bronchiolitis (RB) is a medical condition found in cigarette smokers that does not have any noticeable symptoms. It is characterized by the buildup of macrophages that are tan or yellow in color in the lumens of bronchioles (9). This accumulation is accompanied by chronic inflammation and fibrosis that extends from the respiratory bronchioles to the alveolar walls. When RB is accompanied by clinical evidence of ILD, it is referred to as respiratory bronchiolitis-interstitial lung disease (RB-ILD), which is a distinct form of ILD. The difference between RB and RB-ILD is mainly based on the presence of clinical evidence of ILD, such as symptoms, chest imaging abnormalities, and pulmonary function test results, rather than the extent of fibrosis in the alveolar walls as observed through histopathology (221). A person is usually suspected of having RB-ILD if they have non-specific respiratory symptoms like dyspnea or cough and an abnormal chest radiograph (222). Findings on HRCT are non-specific and include air entrapment, centrilobular nodules, and diffuse or patchy ground-glass opacities. The diagnosis of RB-ILD may be made based on a firm clinical suspicion, compatible pulmonary function test and HRCT results, and mild respiratory impairment (223, 224). Invasive tests may not be necessary if a patient’s condition stabilizes or improves after quitting smoking. Lung biopsy is frequently performed to corroborate the diagnosis and rule out other treatable interstitial lung diseases in cases of more serious or progressive respiratory impairment despite smoking cessation. The most crucial diagnostic and therapeutic stage is giving up smoking and avoiding exposure to cigarette smoke (222, 225).

### 3.7. Radiation-induced lung injury

Radiation Pneumonitis (RP), the initial stage of radiotherapy-induced lung injury (RILI), is defined by lung tissue inflammation brought on by radiation exposure. Radiation Fibrosis (RF), the second stage of RILI, is a clinical condition brought on by persistent pulmonary tissue damage. Currently, a diagnosis is usually made by excluding other potential causes using clinical examination and radiological imaging. Pulmonary function tests are helpful in determining the state of the patient’s lung function and in identifying any possible side effects or toxicity during radiotherapy. Although systemic corticosteroids are frequently used to treat pneumonitis complications, their use needs to be standardized and taken into consideration for prophylactic reasons due to the potentially fatal consequences of this adverse event (226).

## 4. Idiopathic lung diseases

While the etiology of immune-mediated lung disorders is not clearly defined, most of these disorders could be classified under idiopathic lung disease. This review will focus on IPF, the prototype of idiopathic lung diseases where lung injury leads to fibrosis.

### 4.1. Idiopathic pulmonary fibrosis (IPF)

Idiopathic pulmonary fibrosis, the most common type of spontaneously occurring diffuse parenchymal lung disease, is a chronic progressive and eventually lethal disease with a poor prognosis characterized by irreversible diffuse lung fibrosis and impaired lung function likely caused by a multitude of cycles of epithelial cell injury and uncontrolled repair (12, 227). IPF’s histopathological features include aberrant mesenchymal cell proliferation, various degrees of fibrosis, overproduction, and disordered collagen and extracellular matrix deposition (228). Furthermore, the pathogenesis of IPF is complex and not fully known; however, several likely contributors have been recognized in the literature; injured alveolar epithelial cells secrete growth factors, especially TGF-β, which help in the recruitment of fibroblasts and subsequent differentiation into myofibroblasts expressing features of both fibroblasts and smooth muscle cells. The myofibroblasts cause excessive collagen and extracellular matrix (ECM) deposition leading to scar formation and distortion of the alveolar structure. It is possible that multiple micro-injuries to alveolar epithelial cells induce a fibrotic environment (229). Alpha-smooth muscle actin (SMA), which is expressed by myofibroblasts, helps to distinguish them from smooth muscle cells and fibroblasts (230). Myofibroblasts secrete collagen after being recruited to the lungs or differentiating from local fibroblasts. Collagen accumulates as a result of an imbalance between interstitial collagenases and their tissue inhibitors (231).

Over the past decade, research efforts regarding immunity and IPF have reached important milestones. IPF involves both innate and adaptive inflammatory processes (205). Since neutrophils are important in the acute phase of inflammation, their aggregation in response to lung injury may intensify tissue remodeling and fibrosis, presumably *via* neutrophil elastase. B-cells build up in the lungs of people with IPF, and humoral autoimmunity against epithelial auto-antigens can fuel and sustain persistent inflammation there (205). In addition, T-cells have been shown to be involved in IPF. The Th2 cytokines IL-4 and IL-13 are extremely pro-fibrotic, whereas the Th1 cytokines IFN- and IL-12 prevent the development of tissue fibrosis. Th1 and Th2 cytokines play conflicting roles in fibrosis (232).

The risk of IPF is significantly increased by a single nucleotide polymorphism (rs35705950) in the promoter region of MUC5B (233). MUC5B is a gene that codes for mucin 5B, a glycoprotein important for innate immune responses against bacterial and airway clearance. Some researchers have proposed that abnormal mucociliary clearance may cause changes in the lung microbiota and innate immune responses that support IPF. However, the exact mechanism relating to mucin 5B overexpression and IPF risk is yet to be known (195, 234).

#### 4.1.1. Clinical manifestations

Patients usually present with worsening cough, dyspnea, and impaired quality of life. The diagnostic approach initially involves the exclusion of other interstitial lung diseases or overlapping conditions such as hypersensitivity pneumonitis, pulmonary sarcoidosis, or an underlying autoimmune disease. IPF demonstrates “honeycombing” on high-resolution CT scan (subpleural cystic airspaces with well-defined walls). In addition, lung biopsy may be considered in some patients (13, 235). IPF is frequently misdiagnosed, and is improperly treated with immunosuppressive medicine. Recent treatments can halt the progression of the disease (235).

#### 4.1.2. Treatment

Results from previous negative landmark studies imply that the inflammatory changes found in IPF happen independently of the main fibrotic remodeling mechanism. Nevertheless, current medications (such as pirfenidone and nintedanib) or new medications that have shown promise in Phase-II trials for IPF (like PMR151 and GLPG1690) also control inflammatory processes (205). It is crucial to understand that treating lung injury frequently requires treating the inflammation (e.g., infections, physical trauma). Given that immune cells constitute a typical component of the human lungs’ normal structure and operation, this problem may be particularly relevant to inflammatory illnesses of the respiratory system (236). Consequently, there is a critical need for stratified medicine based on genomes, biomarkers, and also inflammatory profiles to identify IPF patients who may benefit from combining standard-of-care “anti-fibrotic” medication with co-treatment with anti-inflammatory/immunomodulatory therapy. For IPF patients, this precision medicine should result in accurate health advice, a precise diagnosis, and a unique treatment strategy (205).

Recent studies have shown that most Interleukin 17 (IL-17) isoforms are involved in acute and chronic inflammation *via* innate and adaptive immunity (237). Cipolla et al. (238) reported that Interleukin 17A (IL-17A) and complement (C’) activation play an important role in the pathogenesis of IPF. IL-17A activates profibrotic signaling pathways and C’ by regulating mRNA and protein expression of the C’ components (238). Consequently, C3a, C5a, and TGF-β mediate alveolar epithelial injury *via* p38MAPK activation (239). Although there is currently no direct evidence that IL-17F contributes to the development of IPF, these findings on IL-17A and inflammatory responses imply that IL-17F may be a useful therapeutic target (237).

Pentraxin 2, also known as serum amyloid P, can prevent the development of fibrocytes that promote fibrosis and inflammatory macrophages. Patients with IPF have lower levels of Pentraxin 2 in their plasma compared to healthy individuals (240). In a phase 2 trial, 111 patients with IPF received either intravenous recombinant human Pentraxin 2 (at a dose of 10 mg/kg) or placebo every 4 weeks for 24 weeks. Most of the patients were already being treated with an anti-fibrotic agent. The Pentraxin 2 group had a slower decline in FVC compared to the placebo group (241). The most common side effects were cough and fatigue. In a follow-up study lasting 76 weeks, patients who were switched from placebo to Pentraxin 2 showed a slower decline in lung function and distance walked in 6 min, which was similar to the initial study (242). These findings were consistent even after 128 weeks (243). Pentraxin 2 was generally well-tolerated by patients.

Phosphodiesterase 4 (PDE4) inhibitor (BI 1015550), which prevents the degradation of cyclic adenosine monophosphate, has antifibrotic and immunomodulatory effects (244). In a placebo-controlled trial to test the efficacy of BI 1015550 in preventing a decline in lung function in patients with idiopathic pulmonary fibrosis. The use of BI 1015550, either alone or in combination with an antifibrotic agent, was successful in preventing a decrease in lung function (245).

In a phase IIb randomized clinical study to assess the safety and efficacy of anti-αvβ6 monoclonal antibody (BG00011). The results did not support the ongoing clinical advancement of BG00011 (246). Additional investigation is necessary to pinpoint better therapeutic methods that can alter the inflammatory and fibrotic pathways in IPF.

## 5. Miscellaneous lung diseases

### 5.1. Sarcoidosis

Sarcoidosis is a multisystem inflammatory disorder of unknown etiology that can affect virtually any organ system but classically involves the lungs and hilar lymph nodes. At the center of sarcoidosis pathogenesis is the non-caseating granuloma, a cellular aggregation orchestrated by a complex ensemble of innate and adaptive immune cells and inflammatory mediators. The perplexing interplay of these immune components is yet to be fully delineated and is of prodigious value in revealing therapeutic avenues for sarcoidosis management (247, 248).

The inciting event in sarcoidosis pathogenesis is thought to be exposure to a yet unidentified antigen detected by antigen-presenting cells (APCs). Putative antigens may be broadly classified as microbial (e.g., mycobacteria and propionibacteria) or non-microbial (e.g., autoantigens such as vimentin, or exogenous antigens such as occupational exposures) (249). The antigen is delivered *via* dendritic cells and presented *via* major histocompatibility complex class II molecules to T cells, promoting their differentiation into various T-cell subsets, including T helper (Th) 1 cells. Early phases of sarcoidosis are characterized by a Th1-promoting milieu of chemokines such as IL-2 and IFN-gamma, which led to sarcoidosis being classically viewed as a Th1-polarized disease (250). Th1 cells elaborate cytokines, including IFN-gamma, which primarily serve to activate macrophages to ingest or destroy the target antigen and amplify the granulomatous response. More recently, however, a shift in the understanding of sarcoidosis pathogenesis occurred with recognition of Th17 cells as new players in the disease. Recent studies have suggested that Th17.1 cells to the pre-dominant source of IFN-gamma in sarcoidosis bronchoalveolar lavage fluid, putting into question the classical Th1 paradigm (251). Another contributing factor to sarcoidosis immunopathogenesis is believed to be an imbalance between the immunosuppressive effects of regulatory T (Treg) cells and the proinflammatory effects of other T-cell subsets (252) interest in the Treg population of cells is growing, and whether disease activity may correlate with the balance between Treg cell activity and other T-cell subset activity would be of great value to the sarcoidosis community.

Although much of sarcoidosis pathogenesis focuses on T-cell-mediated immunity, humoral mechanisms have been suggested to play a role on the basis of several observations, including hypergammaglobulinemia, autoantibodies, circulating immune complexes (253–255). Nevertheless, anti-B-cell therapy with rituximab has shown conflicting results in refractory disease, and further studies are certainly needed to elucidate the true significance of humoral immunity in sarcoidosis immunopathogenesis (256–258). Additionally, the innate arm is being increasingly recognized as an important contributor to sarcoidosis pathogenesis, with derangements in toll-like receptor (TLR) and nucleotide oligomerization domain receptor (NLR) signaling being implicated (249).

Progression to fibrotic pulmonary sarcoidosis is challenging to understand given the pre-dominance of the antifibrotic Th1 cytokine, IFN-gamma, in the milieu of sarcoidosis granulomas. It has therefore been postulated that persistent inflammation may induce a switch from an antifibrotic Th1-predominant state to a profibrotic Th2-predominant state, transitioning into fibrotic pulmonary sarcoidosis (259). Additionally, Treg cells may acquire the capacity to inhibit the Th2 cells and reduce fibrosis (260). Decreased Treg cell numbers in bronchoalveolar lavage fluid, and Treg cell dysfunction have been observed in sarcoidosis and may promote fibrosis (261, 262). Roles for other cells, including Th17 cells and macrophages may also contribute (263). Finally, genetic polymorphisms in various genes, such as TGF-β and GREM1, have also been linked to fibrotic pulmonary sarcoidosis (263).

#### 5.1.1. Clinical manifestations

Sarcoidosis can affect virtually any organ system of the body, but pulmonary involvement is characteristic and occurs in up to 90% of cases (264). Bilateral hilar lymphadenopathy detected incidentally on chest radiography is a typical scenario in an asymptomatic patient and confers an excellent prognosis, with the majority of patients achieving remission within 3 years following the diagnosis (265). In the presence of concurrent parenchymal lung disease which commonly appears in the form of reticulonodular pulmonary infiltrates, respiratory symptoms of dry cough, dyspnea, and chest pain are possible presentations (265). Long-standing pulmonary disease may progress into pulmonary fibrosis, in which case chronic cough and dyspnea are prominent complaints, alongside potential physical exam findings of clubbing, wheezing and end-inspiratory crackles (266). Regardless of organ involvement, constitutional symptoms including fatigue, fever, and night sweats are relatively common (267, 268). Alternatively, classical sarcoidosis syndromes have been described including Lofgren syndrome (bilateral hilar lymphadenopathy, migratory polyarthritis, and erythema nodosum) as well as Heerfrodt syndrome (uveitis, parotitis, and facial nerve palsy), and the presence of either of these syndromes has substantial diagnostic relevance (265). Pulmonary hypertension is a potential complication of sarcoidosis and incidence rates vary from 5 to 70% of sarcoidosis patients depending on the study population. Sarcoidosis-associated pulmonary hypertension (SAPH) patients are almost invariably dyspneic and have a reduced 6-min walk distance when screened for SAPH. SAPH is a significant cause of morbidity and mortality in sarcoidosis patients and confers a poorer prognosis (266). Other pulmonary pathologies in sarcoidosis include pleural disease in the form of pleural effusion or pneumothorax but are relatively uncommon findings in sarcoidosis (264, 266). Extrapulmonary sarcoidosis may coexist with pulmonary disease or occur in isolation, and can involve the skin (e.g., erythema nodosum, lupus pernio), eyes (e.g., uveitis), musculoskeletal system (e.g., arthritis), and nervous systems (e.g., facial nerve palsy, meningitis), heart (e.g., arrhythmias, heart failure) among others (269).

The diagnosis of sarcoidosis is often challenging and emerges in the context of clinical, radiographic, and histopathological evidence of the disease, all in the absence of an alternative explanation for the clinical findings (270). This often necessitates comprehensive evaluation in order to eliminate other sarcoidosis mimics. Chest X-rays are the initial imaging modality of choice and aid in thoracic sarcoidosis staging. Typical findings include bilateral hilar lymphadenopathy and/or pulmonary infiltrates. Computed tomography scans are usually sought for further evaluation of the disease as they demonstrate superior sensitivity in detecting lymphadenopathy and parenchymal lung changes, in addition to better characterizing the presence of pulmonary fibrosis as compared to chest radiographs (265, 271). An absolute diagnosis in many cases, however, calls for a tissue biopsy from the most accessible site. This would ideally be from a cutaneous lesion, but in the absence of peripheral involvement, endobronchial or transbronchial biopsy are the gold standard. Demonstration of non-caseating granulomatous inflammation is the hallmark pathology of sarcoidosis. Laboratory evaluation is complementary and may assist in monitoring disease activity. Classic laboratory findings include hypercalcemia, elevated ACE levels, serum soluble interleukin-2 receptor, rheumatoid factor and elevated inflammatory markers (270, 272).

#### 5.1.2. Treatment

The mainstay of management of pulmonary sarcoidosis remains to be oral glucocorticoid therapy. Immunosuppressive therapy with methotrexate, azathioprine, and leflunomide are second-line options used in glucocorticoid-resistant disease, or as steroid-sparing therapy in those who cannot tolerate steroids or need long-term management. Those who are also refractory to the aforementioned agents may benefit from anti-TNF-alpha therapy with infliximab or adalimumab which are considered third-line options for sarcoidosis (273–276). Numerous other therapies remain investigational, including rituximab, cyclophosphamide, and tocilizumab (256–258, 277). End-stage fibrotic disease is not responsive to immunosuppressive therapy and in such cases, lung transplantation may be the sole option (278, 279). Antifibrotic therapy for progressive pulmonary fibrosis in sarcoidosis remains investigational. Nintedanib was shown to slow disease progression in a recent report, and other antifibrotic agents such as pirfenidone are also under investigation (266).

### 5.2. Post-lung transplant ILD

Post-lung transplant immune-mediated ILD is a condition that can present as either a recurrent episode of the primary disease or as a *de novo* disease after lung transplantation. Due to the rarity of cases, the pathogenesis of recurring or *de novo* ILDs after lung transplantation remains incompletely understood. However, it is crucial for clinicians to be aware that various subtypes of ILD might reoccur after a transplant and to consider this diagnosis in situations with deteriorating respiratory symptoms. Further research is required to gain comprehensive knowledge of the factors that may contribute to the development of ILD after transplantation.

The most common clinical presentation of this condition is the deterioration of respiratory function that requires clinical management or intervention, which can frequently be mistaken for rejection episodes and should be considered by physicians following up on patients despite being relatively rare compared to rejection. To confirm the cause of the sudden deterioration in clinical manifestations, lung biopsies may be necessary. Histopathological examination of lung specimens from patients with post-transplant immune-mediated ILD often reveals one of the patterns of a subtype of ILDs, as well as infiltration of immune cells and fibrotic changes (280).

Lung transplantation can improve quality of life and survival for patients with terminal lung diseases that are resistant to other treatment options. However, in rare cases, primary diseases, including various subtypes of interstitial lung disease (ILD), have been reported to recur after transplantation (280). Collins et al. (281) reported 15 occurrences of primary disease recurrence among 1,394 lung transplant recipients at six medical centers.

Following lung transplantation, sarcoidosis was the most commonly reported cause of recurrence of the primary disease (280–282). Schultz et al. (283) found that about 30% of lung transplant recipients with sarcoidosis experienced recurrence of sarcoid granulomas. Ionescu et al. (284) employed DNA analysis methods and suggested that the origin of recurrent granulomas in the graft is associated with the recipient. Banga et al. (285) found that 7 out of 30 cases experienced recurrence of sarcoidosis over an 18-year period in a single center and determined that the presence of granulomas on explanted lungs was the sole predictor of recurrence Additionally, they observed that sarcoidosis recurrence did not appear to affect 1–5-year survival rates. Although the recurrence of granulomas in transplanted lungs is generally not associated with significant deterioration in allograft function or patient survival, it is important for clinicians to consider arranging appropriate surveillance strategies such as biopsy and high-resolution computed tomography during the post-transplant follow-up period to monitor for the recurrence of sarcoidosis (282).

Idiopathic interstitial pneumonia, including desquamative interstitial pneumonia (DIP), NSIP, has been shown to recur after lung transplantation, with varying time frames reported in the literature (280). King et al. (286) reported a case of DIP recurrence 1 month after transplantation in a 50-year-old female patient, leading to Cytomegalovirus and Nocardia infections, respiratory failure, and ultimately, mortality 8 months after transplantation despite aggressive treatment with high-dose steroids, antibiotics, and mechanical ventilation Verleden et al. (287) reported a case of DIP recurrence in a 51-year-old male patient more than 1 year after his lung transplantation. The patient fully recovered after a course of corticosteroid therapy and a gradual decrease in dosage. No symptoms of dyspnea or coughing were reported for at least 2 years following treatment until the patient was lost to follow-up (287). Bhatt et al. (288) reported a case of a 42-year-old woman who experienced a decline in respiratory function 8 weeks after receiving a bilateral lung transplant for NSIP. Biopsy findings at 6 months post-transplant revealed acute rejection with mixed desquamative and NSIP findings, similar to the patient’s pre-transplant pathology. The study also observed that recipient macrophages began accumulating in the lungs as early as 2 months after transplantation and continued to build up, contributing to the development of interstitial fibrosis.

Connective tissue disease-associated interstitial lung diseases (CTD-ILDs) have been reported to recur or occur *de novo* following lung transplantation. Arboleda et al. (289) described a case of a 15-year-old female patient who received a bilateral lung transplant for polymyositis-related UIP and developed recurrent UIP 8 months post-transplant, ultimately leading to respiratory failure and death 16 days after receiving mechanical ventilation and extracorporeal membrane oxygenation (ECMO) treatment. A post-mortem investigation of the graft revealed advanced pulmonary fibrosis with UIP, indicating a recurrence of the primary disease. Scallan et al. (290) described a case of a 51-year-old female patient who developed recurrent idiopathic fibrotic non-specific interstitial pneumonia (iNSIP-F) and new onset antisynthetase syndrome (anti-SS) 30 months after undergoing bilateral lung transplantation. As of the time this case was reported in 2020, the patient had been 10 years post-transplant and continued to have clinical and serologic features of anti-SS with slowly progressive fibrotic changes, and had also developed pulmonary hypertension that responded to treatment with sildenafil. A meta-analysis of outcomes after lung transplantation found that non-myositis CTD-ILDs had a similar survival rate to IPF, while myositis CTD-ILDs were associated with a poorer survival outcome (291).

## 6. Conclusion

Immune-mediated lung diseases are a heterogeneous group of disorders that pose a diagnostic and therapeutic challenge. Timely diagnosis of immune-mediated lung diseases is important. Moreover, frequent follow-up to assess disease progression is necessary. A personalized approach to treatment is required given the heterogeneity of the presentation and overlap among multiple disorders. CS remains the mainstay as initial treatment. However, the dose and duration vary depending on the primary disease, systemic involvement, inflammatory lung changes and the presence or absence of lung fibrosis. In patients who experience CS toxicity or where CS lacks efficacy, alternatives may be helpful. In cases where there may be concomitant fibrosis, anti-fibrotic agents may be considered. There are no standardized management guidelines, thus a collaborative treatment team should consider a personalized approach according to the underlying lung disease, rate of disease progression, and disease severity. Special attention should be paid to the risk of infection and to proper vaccination. Advanced research technologies combined with artificial intelligence will allow us to identify potential therapeutic targets for inflammatory and fibrotic phenotypes. Furthermore, it will allow us to design translational clinical trials that will offer therapeutic options and may potentially decrease the progression to severe lung disease.

## Author contributions

JS, NWS, and NS were built the table. NWS designed the figure. All authors contributed to the scientific writing of the manuscript and approved the submitted version.
